# Utility of a Recombinant HSV-1 Vaccine Vector for Personalized Cancer Vaccines

**DOI:** 10.3389/fmolb.2022.832393

**Published:** 2022-01-26

**Authors:** Ifeanyi Kingsley Uche, Brent A. Stanfield, Jared S. Rudd, Konstantin G. Kousoulas, Paul J. F. Rider

**Affiliations:** ^1^ Division of Biotechnology and Molecular Medicine Department of Pathobiological Sciences, School of Veterinary Medicine, Louisiana State University, Baton Rouge, LA, United States; ^2^ Department of Pathobiological Sciences, School of Veterinary Medicine, Louisiana State University, Baton Rouge, LA, Untied States

**Keywords:** HSV-1, VC2, oncolytic virotherapy, herpes, cancer, personalized vaccine

## Abstract

Current approaches to cancer immunotherapy include immune checkpoint inhibitors, cancer vaccines, and adoptive cellular therapy. These therapies have produced significant clinical success for specific cancers, but their efficacy has been limited. Oncolytic virotherapy (OVT) has emerged as a promising immunotherapy for a variety of cancers. Furthermore, the unique characteristics of OVs make them a good choice for delivering tumor peptides/antigens to induce enhanced tumor-specific immune responses. The first oncolytic virus (OV) approved for human use is the attenuated herpes simplex virus type 1 (HSV-1), Talimogene laherparepvec (T-VEC) which has been FDA approved for the treatment of melanoma in humans. In this study, we engineered the recombinant oncolytic HSV-1 (oHSV) VC2-OVA expressing a fragment of ovalbumin (OVA) as a fusion protein with VP26 virion capsid protein. We tested the ability of VC2-OVA to act as a vector capable of stimulating strong, specific antitumor immunity in a syngeneic murine melanoma model. Therapeutic vaccination with VC2-OVA led to a significant reduction in colonization of tumor cells in the lungs of mice intravenously challenged B16cOVA cells. In addition, VC2-OVA induced a potent prophylactic antitumor response and extended survival of mice that were intradermally engrafted with B16cOVA tumors compared with mice immunized with control virus.

## Introduction

It is currently understood that cancers result from individual cellular transformation events resulting in genetically and phenotypically unique tumors even within the same tissue environment (Al-Hajj and Clarke, 2004). This is problematic for the development of therapeutic or prevention strategies that seek to treat patient populations based on common features of tumors such as their tissue of origin. It is not surprising therefore, that current drugs for treating cancer only work for a small number of patients with a given cancer type (Chiriva-Internati and Bot, 2015). Thus, a personalized medicine approach is needed to tailor immunotherapies that are based on identifiable characteristics of patient-specific tumors.

Current molecular diagnostics, including genomic and proteomic tools, allow us to employ greater precision in the design and delivery of anti-cancer treatments and therapies (Krzyszczyk et al., 2018; Nassar et al., 2020; Rodriguez et al., 2021). These tools avail physicians and scientists with incredible amounts of information regarding mutations that are unique to a particular patient. Examples include the identification of druggable pathways that result from such mutations, or the targeting novel kinase fusions in various cancer types (Stransky et al., 2014; Krzyszczyk et al., 2018). Additionally, these tools can be used to identify so-called tumor associated antigens (TAAs) (Hu et al., 2018). TAAs are the protein products of mutated genes that are not found in the proteome of healthy, non-transformed cells. TAAs result from genetic mutations and are unique to specific patients. As the immune system has evolved to discriminate self from non-self and eliminate non-self, TAAs can be used to target host immune responses to cells that bear these TAAs (Hu et al., 2018). This approach results in a “personalized” therapy.

Personalized therapies include CAR-T-cells, bispecific antibodies, and several approaches to induce *de novo* TAA specific immune responses via mRNA and vaccines, peptide vaccines and viral vectored TAAs (Slingluff et al., 2007; Kantoff et al., 2010; Rittig et al., 2011; Hu et al., 2018; Krzyszczyk et al., 2018). While there are currently no FDA-approved TAA vaccines, many groups have reported successes in clinical and pre-clinical work, and there is a great deal of interest and activity in this area (Goldman and DeFrancesco, 2009).

Regarding viral vectored TAA vaccines, there are several approaches currently being pursued (Holay et al., 2017). Viral vectors must possess both safety as well as immunogenicity. There are several attributes of human herpesviruses that inform their use as vaccine vectors: 1) they can infect humans in the presence of a significant anti-viral host response, 2) their relative safety, 3) their large size allowing the insertion of multiple transgenes within their viral genomes without compromising viral replication and infectivity, 4) the ease of genetic manipulation allowing the rapid and efficient generation of recombinant viruses, 5) the availability of anti-herpes drugs to control potential breakthrough infections, and 6) availability of a significant body of knowledge regarding the molecular biology of human herpesviruses which allows targeted manipulation of the viral genome to avoid downregulation of specific immune responses while augmenting others (Uche et al. 2021).

Our laboratory has developed the HSV-1 vaccine vector strain, VC2 (Stanfield et al., 2014). Specific mutations in VC2 glycoprotein K (gK) and the UL20 membrane protein abrogate its ability to infect neurons and establish latent infection (Jambunathan et al., 2015). The inability to establish latent infection and subsequently reactivate, is a unique safety feature. We have shown in several animal trials, including mouse, guinea pig, and non-human primate studies, that VC2 is a safe and immunogenic vaccine strain (Stanfield et al., 2017,., 2018; Naidu et al., 2020). We have further shown that VC2 confers protection of against lethal HSV genital and ocular infection (Stanfield et al., 2014; Bernstein et al., 2019; Naidu et al., 2020).

Previously, we reported that VC2 induced potent anti-tumor immune responses when administered intratumorally to melanoma tumors in immunocompetent mice (Uche et al., 2021a). Herein, we evaluated the utility of VC2 as a vaccine vector for prophylactic and therapeutic anti-cancer applications. To this end we generated the recombinant virus, VC2-OVA, expressing the immunogenic OVA peptide fused in-frame to the amino-terminus of the VP26 viral capsid protein. This allows maximal expression of the immunogen in infected cells, as well as its incorporation into the virion particle. We evaluated the efficacy of VC2-OVA in a syngeneic mouse model of melanoma. Specifically, we took advantage of widely used experimental mouse models of melanoma that express ovalbumin: B16cOVA (melanoma). Finally, we evaluated the differences between intradermal, subcutaneous and intramuscular routes of vaccination with VC2-OVA. Vaccination with VC2-OVA prevented the growth of engrafted tumors in both prophylactic and therapeutic settings. Importantly, our results show that the specific route of vaccination had a profound impact on the success of prophylactic treatment. Taken together these data demonstrate the potential of the VC2-vectored approach for personalized anti-cancer therapeutics.

## Materials and Methods

### Animals

Four-to five-week-old female C57BL/6J mice were purchased from the Jackson Laboratory (Bar Harbor, ME). All mice were maintained in pathogen-free facilities. Protocols involving animals were reviewed and approved by the Louisiana State University Institutional Animal Care and Use Committee (IACUC), and all animal experiments were performed in accordance with the protocols.

### Construction of the VC2-OVA Virus

The bacterial artificial chromosome (BAC) plasmid VC2 was used to construct VC2-OVA as previously described (Stanfield et al., 2014). High-efficiency markerless DNA manipulation of VC2 was achieved using two-step red-mediated recombination (Karstentischer et al., 2006). Oligonucleotides used in the construction of the recombinant virus are presented in Supplementary Table S1. Recombinant HSV-1 was recovered after BACs were transfected into Vero cells using Lipofectamine according to the manufacturer’s protocol. DNA was extracted from viral stocks, and VP26 was sequenced to ensure the presence of the desired mutation. Virus for experimentation was purified as follows: Vero cells were infected and at full cytopathic effect (CPE), cells and supernatant were harvested. The cellular portion was separated from the supernatant by centrifugation at 4,000 RPM for 10 min. The supernatant was removed and the cell pellet was lysed by freezing and thawing of the pellet 3 times. The supernatant was added to the lysed cellular portion followed by a second round of centrifugation at 4,000 RPM for 10 min. The supernatant was aliquoted and titered to perform experiments.

### Western Blot Analysis

Vero cells were uninfected or infected at an MOI 1 with either VC2 or VC2-OVA for 24 and 48 h in a six well plate. Adherent cells were washed 3x in PBS followed by lysis in 200 μl of NP40 lysis buffer with protease/phosphatase inhibitors. Twenty microliters of whole cell lysate were then mixed with Laemmli sample buffer (Bio-Rad) and 1 μl of *β*-mercaptoethanol to a final 1x concentration. These mixtures were then boiled at 100°C for 10 min and cooled on ice before loading into a 12% Mini-PROTEAN TGX precast gel (Bio-Rad) and separated for 1 h at 100 V in 1x Tris-Glysine-SDS buffer (Bio-Rad). Separated protein was then transferred to a nitrocellulose membrane in 1x Tris-Glysine + 20% methanol (Bio-Rad). The membrane was then blocked for 30 min in 5% BSA in PBS-T. Rabbit anti-VP26 (Kind gift from Prashant Desai, Johns Hopkins), was diluted 1:1,000 in 5% BSA PBS-T and applied to the membrane and incubated overnight at 4°C while rocking. The next day, the membrane was then washed 3x with PBS-T and secondary goat anti-Rabbit IgG (Abcam: ab6721) diluted 1:1,000 in 5% BSA PBS-T applied to the membrane and incubated at room temperature for 1 h. The membrane was then washed 3x in PBS-T and visualized using ECL Western Blot Substrate (Pierce) and exposure film.

### Cell Culture

The ovalbumin-expressing B16 melanoma cell line (B16cOVA) was a kind gift from Dr. Timothy N.J. Bullock (University of Virginia, Charlottesville, Virginia, United States). B16cOVA cells were grown in RRPMI 1640 medium (Sigma-Aldrich, St. Louis, MO) supplemented with 10% filtered, heat inactivated fetal bovine serum (Gibco-BRL, Grand Island, NY), 100 μg/ml Primocin (Invivogen, San Diego, CA), plus 10 μg/ml Blastocydin (Invitrogen Life Technologies, Grand Island, NY). African green monkey kidney (Vero) cells were cultured in DMEM containing 10% FBS and 100 μg/ml Primocin.

### Tumor Engraftment and Treatment Regimens

For prophylactic assessment, mice were not treated; intramuscularly; intradermally; or subcutaneously vaccinated with 1 × 10^6^ pfu of VC2 or 1 × 10^6^ pfu of VC2-OVA in volumes of 100 ul. Fourteen days after prime immunization, booster immunizations were administered. Six days post-boost, mice were engrafted with 5 × 10^5^ B16cOVA cells in 100 μl PBS orthotopically in the dermis of the dorsal left dorsal pinna. Tumors were measured approximately every 2,3 days by using a digital caliper when tumors reached 50 to 100 mm^3^. Tumor volumes were calculated by using formula 1/2 (length × width^2^). Tumor bearing mice were euthanized when tumors reached greater than 1000 mm^3^ or when mice were excessively moribund. To assess the therapeutic effect, mice were injected intravenously with 5 × 10^5^ B16cOVA cells in 100 μl PBS, and then intramuscularly; intradermally; or subcutaneously vaccinated the next day for two consecutive days. Mice were sacrificed 3 weeks post engraftment, and lungs were removed and the tumor colonies on the lung surface were counted.

### ELISPOT Assays

One day after boost vaccination, mice were sacrificed, and spleens were removed.

Splenocytes (7.5 × 10^5^) were isolated and cultured overnight with either gB peptide (1 μg/ml) or ovalbumin [OVA257–264 (SIINFEKL)] peptide (1 μg/ml). IFN-*γ*-producing splenocytes were quantified according to the manufacturer’s instructions using an Immunospot (Shaker Heights, OH) murine IFN-*γ* single-color ELISPOT assay.

### Statistical Analysis

All statistical analyses were done using GraphPad Prism nine Software (GraphPad Software, Inc., San Diego, CA). Analysis of data between three or more groups was performed by using one-way ANOVA. Survival data were presented using Kaplan–Meier survival curves and differences among groups were analyzed by the log rank test. A *p*-value of 0.05 or less was considered statistically significant in all analyses herein.

## Results

### Construction and Characterization of Ovalbumin Expressing Virus

**FIGURE 1:**
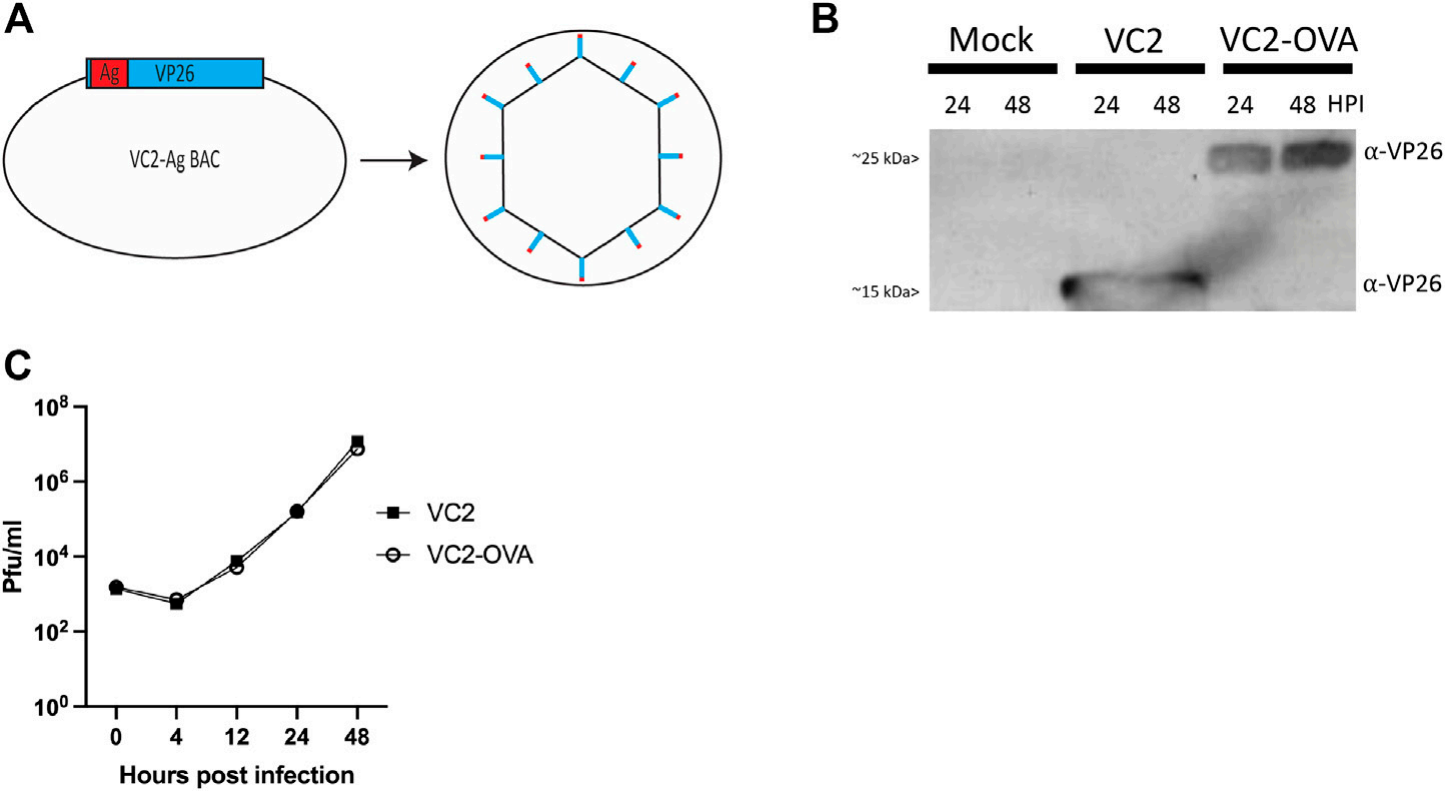
Construction of VC2-OVA virus. **(A)** VC2-OVA. **(B)** Expression of OVA VP26 fusion protein. Vero cells were infected at a multiplicity of infection of 1. Twenty-four and 48 hours post infection, protein lysates were prepared and analyzed by western blot. The blot was stained with antibody to VP26. **(C)** Growth curve of VC2-OVA and parental VC2 viruses. Vero cells were infected at a multiplicity of infection of .01. Supernatants and cell pellets were harvested at indicated times post infection, and plaque assays were performed to determine viral titers.

We wished to fully exploit the potential of viruses to deliver antigen and promote strong, broad, and effective anti-immunogen responses in the host. To this end, we fused the immunogenic portion of chicken egg ovalbumin to VP26, the minor capsid protein of HSV-1 (Figure 1A). Ovalbumin is a common experimental immunogen with an extensive history of use for studying immunogenicity of novel vaccine approaches (Karandikar et al., 2019). VP26 is present at approximately 900 copies in each virion (Kobayashi et al., 2017). This means that in an inoculum of 10^6^ pfu we can deliver nearly 10^9^ OVA-VP26 antigens. However, this extrapolation is likely an underestimation due to a particle to pfu ratio for tissue culture-derived HSV-1 reported to be 100:1 (Mahiet et al., 2012). Further, the fusion of an antigen to the viral particle allows access to the exogenous antigen presentation pathway to promote the development of TH2 responses in addition to traditional TH1 responses to viral vectored antigens. Using BAC mutagenesis, a portion of ovalbumin containing the canonic CD8^+^ peptide (SIINFEKL, OVA_257-264_) was fused to the amino terminus of VP26 to generate VC2-OVA.

To confirm expression of the fusion protein in recovered VC2-OVA, Vero cells were infected at a multiplicity of infection of 1. Twenty-four and 48 hours post-infection, protein lysates were prepared, and a western blot was performed. Using an antibody to detect VP26 we readily observed a protein of the expected size (12kDA (Desai and Person, 1998)) in lysates from cells infected with parental VC2 virus (Figure 1B). However, in lysates from cells infected with VC2-OVA we observed a protein at an apparent molecular mass of approximately 25 kDa, the expected molecular weight of the VP26-OVA fusion protein (Figure 1B).

To determine any effect of fusing ovalbumin to VP26 on viral replication we performed a multi-step growth curve comparing parental VC2 virus and VC2-OVA. Vero cells were infected at an MOI of .01 and cells were harvested at 0, 4, 12, 24 and 48 h post infection. Standard plaque assays were performed to quantify virus in cell lysates. We were unable to identify any difference in viral replication between parental and VC2-OVA viruses (Figure 1C).

### Immunogenicity of VC2-OVA in Mice

**FIGURE 2:**
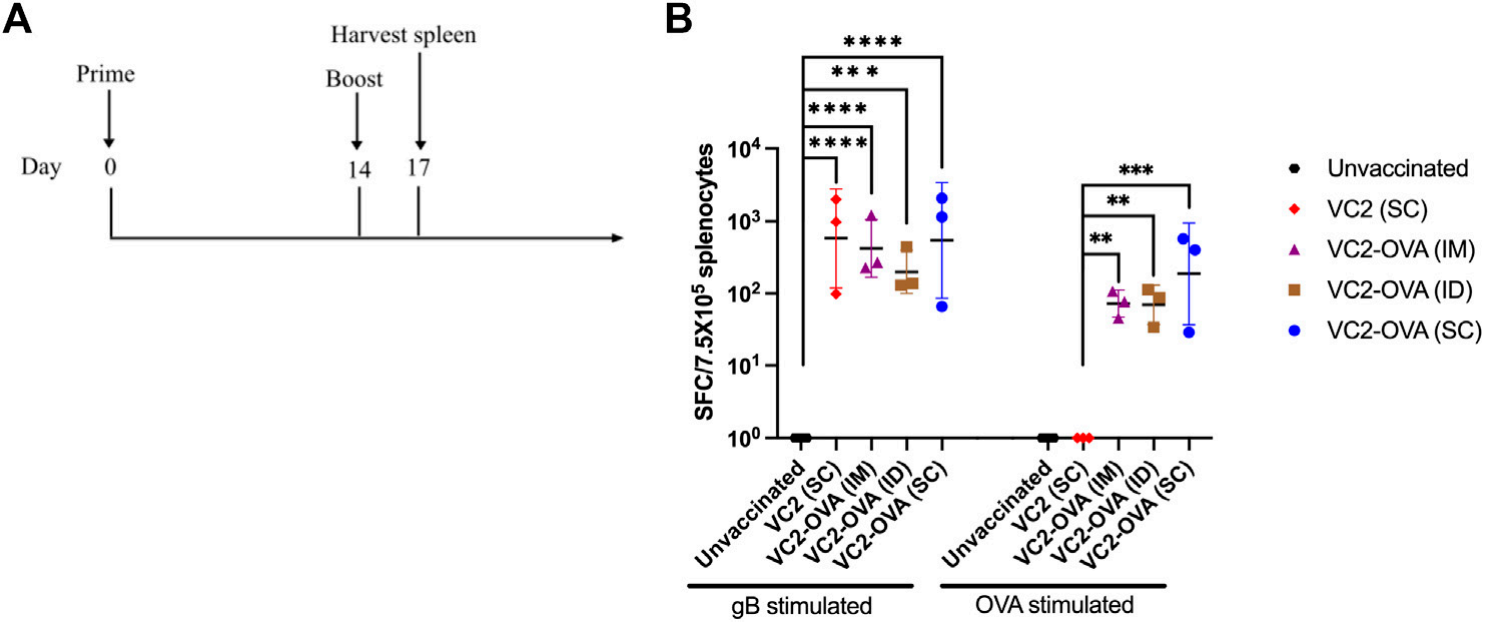
Vaccination with VC2-OVA induces OVA specific T cell response. **(A)** Timeline of treatment regimen. Mice were untreated or prime vaccinated intramuscularly, or intradermally, or subcutaneously with 1 × 10^6^ pfu of VC2-OVA or parental VC2 virus. Fourteen days later, boost immunizations were administered. 72 hours post boost, mice were euthanized, and their spleens were harvested. **(B)** Isolated splenocytes (7.5 × 10^5^) were cultured overnight with either gB peptide (1 μg/ml) or OVA257–264 [SIINFEKL] peptide (1 μg/ml), and IFN-*γ* producing cells were quantified by ELISPOT assay. N = 3 mice per group. Data were analyzed using one-way ANOVA. **, *p <* 0.01, ***, *p <* 0.001.

To test the ability of VC2-OVA to induce OVA-specific immune responses we vaccinated mice with VC2-OVA. After 14 days, mice received a second vaccination (boost) with VC2-OVA or the parental virus. 72 hours post boost vaccination, mice were sacrificed and splenocytes were harvested (Figure 2A). Splenocytes were incubated with either HSV-1 glycoprotein B peptide or SIINFEKL peptide and ELISPOT analysis was performed. The gB peptide is a dominant CD8^+^ T-cell epitope (Treat et al., 2017) and serves as a positive control. We observed that vaccination with VC2 and VC2-OVA, induced high levels of gB specific immune responses (Figure 2B). However, only in splenocytes from mice vaccinated with VC2-OVA was an OVA specific T cell response detected (Figure 2B). Interestingly, there was no significant difference in these responses induced by the different vaccination routes in those animals.

### Efficacy of VC2-OVA in an Experimental Mouse Model of Melanoma

**FIGURE 3:**
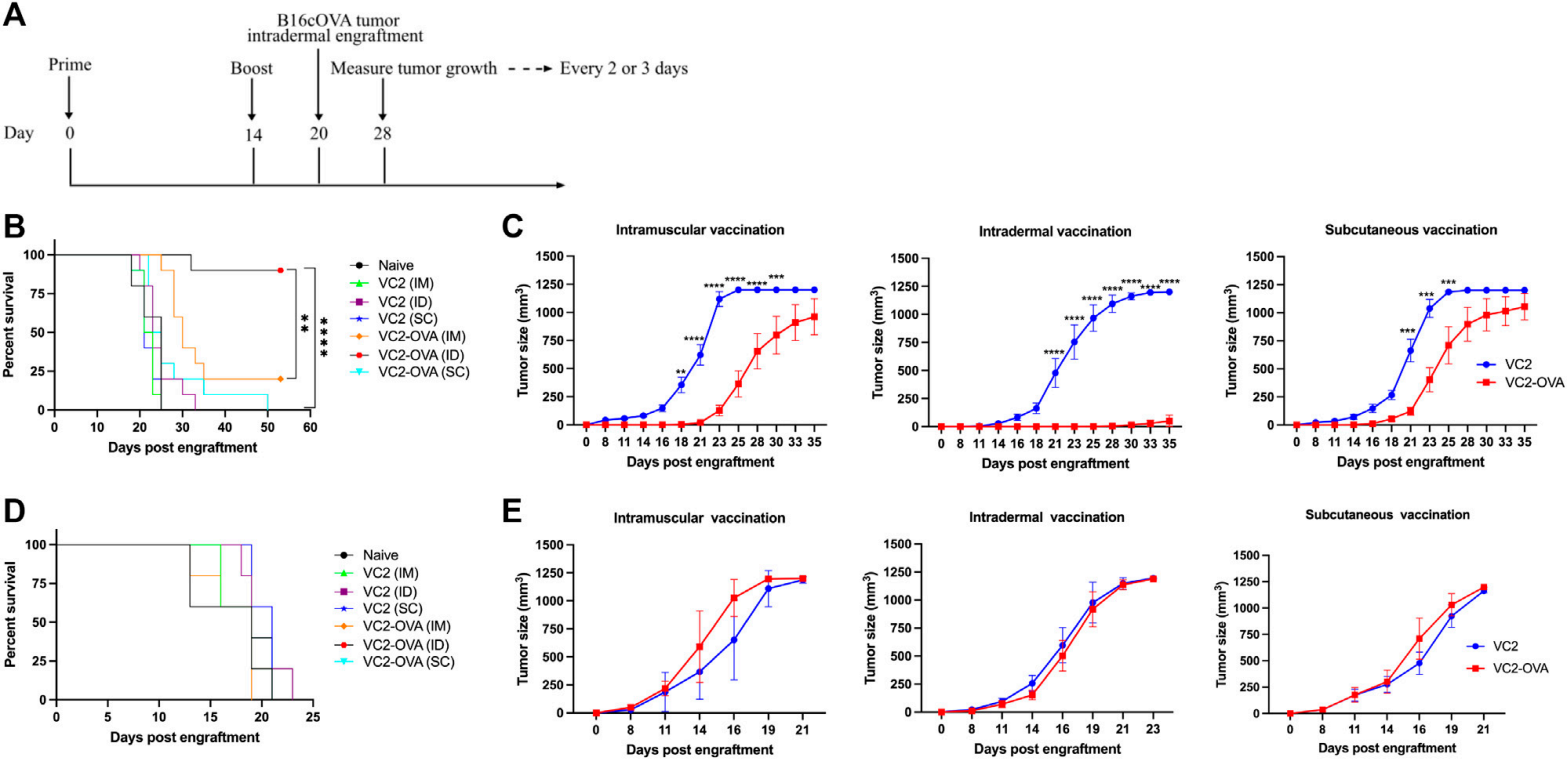
The prophylactic effect of VC2-OVA in B16cOVA tumor model. **(A)** Timeline of treatment regimen. Mice were untreated or prime vaccinated intramuscularly, or intradermally, or subcutaneously with 1 × 10^6^ pfu of VC2-OVA or parent VC2 virus. Fourteen days later, booster immunizations were administered. Six days post vaccination, mice were engrafted with 5 × 10^5^ B16cOVA or B16F10 tumor cells. Mice were observed for tumor growth. Mice were sacrificed when tumors reached greater than 1,000 mm^3^ or when the mice became excessively moribund. Kaplan-Meier survival curves **(B, D)**. Tumor volume and growth rates was measured every 2,3 days **(C, E)**. N = 5–10 mice per group. **, *p <* 0.001, ***, *p <* 0.001, ****, *p <* 0.0001.

We have previously shown the efficacy of parental VC2 in intratumoral treatment of mice engrafted with modified B16F10 melanoma (Uche et al., 2021a). In those previous experiments, we achieved between 50 and 80% cure rates. To investigate whether the expression of a tumor-associated surrogate protein can be used to augment anti-tumor immune response, we employed B16F10 cells which express OVA in conjunction with the VC2 OVA expressing virus administered by direct inoculation into engrafted B16cOVA tumors. There were no significant differences between parental VC2 and VC2-OVA (data not shown). We believe that this is due to the very high cure rate with VC2 treatment that could not be significantly augmented by the presence of the OVA antigen. Next, we tested the efficacy of VC2-OVA in preventing tumor growth in mice that had been vaccinated before engraftment of B16cOVA tumors. The relevance of this approach may be seen in a case where surgical resection of a tumor is followed by vaccination against recurrence. In these experiments we compared the efficacy of VC2-OVA using three distinct vaccination routes: intramuscularly (IM), subcutaneously (SC), or intradermally (ID). We chose this approach as recent data suggests that the efficacy of vaccination can be dependent on the route of vaccination (Zhang et al., 2015). Animals were vaccinated twice, 14 days apart, before tumor engraftment 6 days after the second vaccination (Figure 3A). Mice vaccinated with VC2 (regardless of route of vaccination) were sacrificed 35 days post engraftment (Figure 3B). In contrast to mice vaccinated with parental VC2, all mice vaccinated with VC2-OVA before engraftment had significantly increased median survival times. Interestingly, mice vaccinated with VC2-OVA exhibited survival times that were dependent on route of vaccination. Ninety percent of mice that were ID vaccinated before engraftment arrested tumor growth and survived. Twenty percent of mice that were vaccinated IM survived while none of the mice vaccinated SC survived. Tumor growth rates were consistent with the results of survival with few mice vaccinated intradermally exhibiting tumor growth at all while intramuscular vaccination resulted in slower tumor growth rates than subcutaneous vaccination (Figure 3C). For control purposes, we engrafted mice previously vaccinated with either VC2 or VC2-OVA with B16F10 cells which do not express ovalbumin. In these experiments there were no differences in survival times or tumor growth rates, regardless of vaccination with VC2 or VC2-OVA (Figures 3D,E).

**FIGURE 4:**
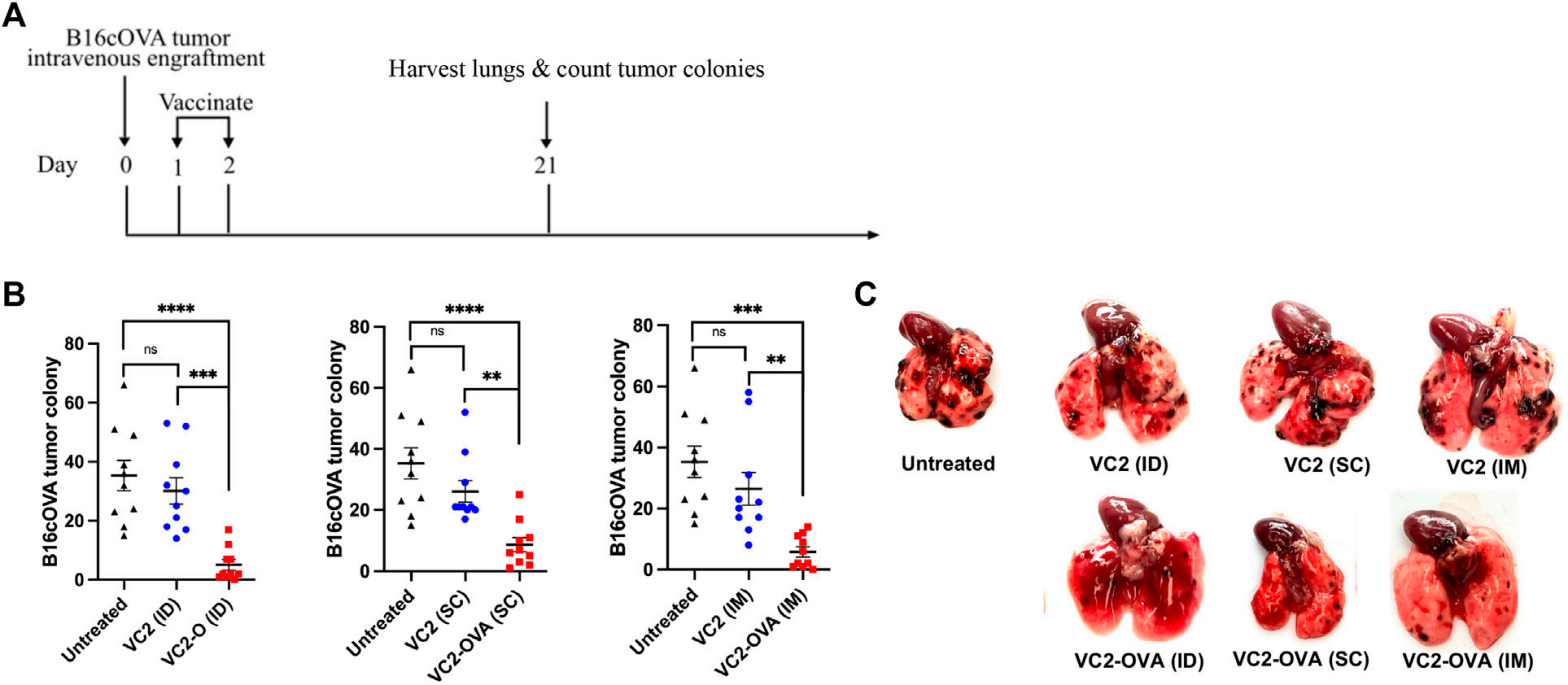
Therapeutic response of VC2-OVA in B16cOVA tumor model. **(A)** Timeline of treatment regimen. Mice were intravenously challenged with 5 × 10^5^ B16cOVA tumor cells. The next 2 days, mice were treated with either VC2-OVA or VC2 through the different vaccination routes. **(B)** Nineteen days post treatment, mice were euthanized and tumor colonies on the lung surface were quantified. **(C)** Representative images of lungs from untreated, VC2, VC2-OVA treated mice 3 weeks postinoculation and quantification of colonization. N = 10 mice per group. Data were analyzed using one-way ANOVA. **, *p <* 0.01,***, *p <* 0.001, ****, *p <* 0.0001, ns = not significant.

Next, we investigated the efficacy of VC2-OVA when used in a therapeutic context, where engraftment preceded treatment. In these experiments, B16cOVA cells were inoculated intravenously. The introduction of these cells intravenously leads to colonization of the lungs by the B16F10 cells resulting in tumors that can be enumerated approximately 3 weeks post engraftment. This approach is a commonly used approach to test intervention strategies for metastasis and the development of systemic anti-tumor immunity. B16cOVA cells were administered intravenously, and mice were treated with either VC2 or VC2-OVA IM, SC, or ID 2 days post tumor administration (Figure 4A). Twenty-one days post engraftment, mice were sacrificed and colonies of B16cOVA cells in the lungs were enumerated (Figure 4B). Mice that were left untreated or treated with VC2 had significantly more tumor colonies in their lungs than mice treated with VC2-OVA (Figures 4B,C).

## Discussion

The identification of safe, and immunogenic vaccine vectors capable of inducing potent immune responses is critical to the development of anti-infectious disease and anti-cancer intervention strategies (Vance et al., 2017). Previously, we demonstrated that the novel HSV-1 (VC2) vaccine vector, can be used to induce potent anti-tumor immune responses in a mouse model of melanoma (Uche et al., 2021b). Herein, we extend our previous findings by demonstrating that VC2 can be readily adapted to promote TAA-specific immune responses capable of extending mouse survival and decreasing tumor growth rates.

Of particular interest is our finding that the route of vaccination was a large factor in the efficacy of treatment. Intradermal route of vaccination proved best in our B16cOVA melanoma model in a prophylactic context. Intramuscular route of vaccination proved to be the least effective in both extending survival and reducing tumor growth rates. It is unclear why intradermal delivery of the virus produced a more efficient vaccination approach. It has been documented that immune responses are affected by the route of vaccination (Belyakov and Ahlers, 2009; Zhang et al., 2015). There are differing reports on whether there is an actual difference in the magnitude or quality of adaptive immune responses generated by differing routes of administration (Ols et al., 2020; Rosenbaum et al., 2021). What makes our study particularly compelling is that we have a functional readout on the route-dependent promotion of anti-tumor responses based on survival and tumor growth rates. Our data strongly suggest that there are significant differences in the outcome of treatment based on the route of administration.

It is important to point out that route of administration is not a one size fits all problem. Likely each route of administration induces specific types of immunity that may be individually suited to protect against different infection and tumor types. Along these lines we note that our studies used two different engraftment sites: intradermal and intravenous. While we saw large differences in route of administration for the intradermally engrafted tumors we didn’t find any difference for the route of administration when tumors were engrafted intravenously. These findings suggest that the route of administration may be an important consideration for infections and tumor types at some sites but not others.

In these experiments we have used an experimental immunogen, OVA, to evaluate the utility of HSV-1 in general, and VC2 specifically, as a vector to deliver tumor associated antigens for treatment of cancer. It is important to note that the clinical utility of our approach will depend on the identification of similarly immunogenic tumor associated antigens in human patients. The identification of such antigens in human tumors is an active area of investigation with encouraging results (Buonaguro et al., 2011; Hu et al., 2018). The identification of such antigens is however fruitless without the development of technologies, such as ours, to deliver TAAs to patients. Future experimentation should therefore focus on using highly immunogenic vectors to target tumor specific TAAs.

In summary, we find that there is significant evidence to pursue viral vectored TAA delivery in general and VC2-derived TAA vaccines specifically. VC2 has proven safe and efficacious as an HSV vaccine in a variety of animal models and preparations are ongoing for a pilot in-human trial. As we have shown that VC2 works very well as an oncolytic virotherapy, we are excited about the prospect of using VC2 as a combination OVT and personalized vaccine for the treatment of human and animal cancers.

## Data Availability

The original contributions presented in the study are included in the article/Supplementary Material, further inquiries can be directed to the corresponding authors.

## References

[B1] Al-Hajj M. Clarke M. F. (2004). Self-renewal and Solid Tumor Stem Cells. Oncogene 23, 7274–7282. 10.1038/sj.onc.1207947 15378087

[B2] Belyakov I. M. Ahlers J. D. (2009). What Role Does the Route of Immunization Play in the Generation of Protective Immunity against Mucosal Pathogens. J. Immunol. 183, 6883–6892. 10.4049/jimmunol.0901466 19923474

[B3] Bernstein D. I. Pullum D. A. Cardin R. D. Bravo F. J. Dixon D. A. Kousoulas K. G. (2019). The HSV-1 Live Attenuated VC2 Vaccine Provides protection against HSV-2 Genital Infection in the guinea Pig Model of Genital Herpes. Vaccine 37, 61–68. 10.1016/j.vaccine.2018.11.042 30471955

[B4] Buonaguro L. Petrizzo A. Tornesello M. L. Buonaguro F. M. (2011). Translating Tumor Antigens into Cancer Vaccines. Clin. Vaccin. Immunol 18, 23–34. 10.1128/cvi.00286-10 PMC3019775 21048000

[B5] Chiriva-Internati M. Bot A. (2015). A New Era in Cancer Immunotherapy: Discovering Novel Targets and Reprogramming the Immune System. Int. Rev. Immunol. 34, 101–103. 10.3109/08830185.2015.1015888 25901856

[B6] Desai P. Person S. (1998). Incorporation of the Green Fluorescent Protein into the Herpes Simplex Virus Type 1 Capsid. J. Virol. 72, 7563–7568. 10.1128/jvi.72.9.7563-7568.1998 9696854 PMC110002

[B7] Goldman B. DeFrancesco L. (2009). The Cancer Vaccine Roller Coaster. Nat. Biotechnol. 27, 129–139. 10.1038/nbt0209-129 19204689

[B8] Holay N. Kim Y. Lee P. Gujar S. (2017). Sharpening the Edge for Precision Cancer Immunotherapy: Targeting Tumor Antigens through Oncolytic Vaccines. Front. Immunol. 8, 800. 10.3389/fimmu.2017.00800 28751892 PMC5507961

[B9] Hu Z. Ott P. A. Wu C. J. (2018). Towards Personalized, Tumour-specific, Therapeutic Vaccines for Cancer. Nat. Rev. Immunol. 18, 168–182. 10.1038/nri.2017.131 29226910 PMC6508552

[B10] Jambunathan N. Charles A. S. Subramanian R. Saied A. A. Naderi M. Rider P. (2015). Deletion of a Predicted β-Sheet Domain within the Amino Terminus of Herpes Simplex Virus Glycoprotein K Conserved Among Alphaherpesviruses Prevents Virus Entry into Neuronal Axons. J. Virol. 90, 2230–2239. 10.1128/JVI.02468-15 26656706 PMC4810717

[B11] Kantoff P. W. Schuetz T. J. Blumenstein B. A. Glode L. M. Bilhartz D. L. Wyand M. (2010). Overall Survival Analysis of a Phase II Randomized Controlled Trial of a Poxviral-Based PSA-Targeted Immunotherapy in Metastatic Castration-Resistant Prostate Cancer. Jco 28, 1099–1105. 10.1200/jco.2009.25.0597 PMC2834462 20100959

[B12] Karandikar S. H. Sidney J. Sette A. Selby M. J. Korman A. J. Srivastava P. K. (2019). New Epitopes in Ovalbumin Provide Insights for Cancer Neoepitopes. JCI Insight 5, e127882. 10.1172/jci.insight.127882 PMC6538383 30869653

[B13] Karstentischer B. von Einem J. Kaufer B. Osterrieder N. (2006). Two-step Red-Mediated Recombination for Versatile High-Efficiency Markerless DNA Manipulation in *Escherichia coli* . Biotechniques 40, 191–197. 10.2144/000112096 16526409

[B14] Kobayashi R. Kato A. Sagara H. Watanabe M. Maruzuru Y. Koyanagi N. (2017). Herpes Simplex Virus 1 Small Capsomere-Interacting Protein VP26 Regulates Nucleocapsid Maturation. J. Virol. 91, e01068. 10.1128/JVI.01068-17 28679756 PMC5571263

[B15] Krzyszczyk P. Acevedo A. Davidoff E. J. Timmins L. M. Marrero-Berrios I. Patel M. (2018). The Growing Role of Precision and Personalized Medicine for Cancer Treatment. Technology (Singap World Sci) 06, 79–100. 10.1142/s2339547818300020 PMC6352312 30713991

[B16] Mahiet C. Ergani A. Huot N. Alende N. Azough A. Salvaire F. (2012). Structural Variability of the Herpes Simplex Virus 1 GenomeIn VitroandIn Vivo. J. Virol. 86, 8592–8601. 10.1128/jvi.00223-12 22674981 PMC3421737

[B17] Naidu S. K. Nabi R. Cheemarla N. R. Stanfield B. A. Rider P. J. Jambunathan N. (2020). Intramuscular Vaccination of Mice with the Human Herpes Simplex Virus type-1(HSV-1) VC2 Vaccine, but Not its Parental Strain HSV-1(F) Confers Full protection against Lethal Ocular HSV-1 (McKrae) Pathogenesis. PLoS One 15, e0228252. 10.1371/journal.pone.0228252 32027675 PMC7004361

[B18] Nassar S. F. Raddassi K. Ubhi B. Doktorski J. Abulaban A. (2020). Precision Medicine: Steps along the Road to Combat Human Cancer. Cells 9, 2056. 10.3390/cells9092056 PMC7563722 32916938

[B19] Ols S. Yang L. Thompson E. A. Pushparaj P. Tran K. Liang F. (2020). Route of Vaccine Administration Alters Antigen Trafficking but Not Innate or Adaptive Immunity. Cel Rep. 30, 3964–3971. 10.1016/j.celrep.2020.02.111 PMC7198771 32209459

[B20] Rittig S. M. Haentschel M. Weimer K. J. Heine A. Muller M. R. Brugger W. (2011). Intradermal Vaccinations with RNA Coding for TAA Generate CD8+ and CD4+ Immune Responses and Induce Clinical Benefit in Vaccinated Patients. Mol. Ther. 19, 990–999. 10.1038/mt.2010.289 21189474 PMC3098631

[B21] Rodriguez H. Zenklusen J. C. Staudt L. M. Doroshow J. H. Lowy D. R. (2021). The Next Horizon in Precision Oncology: Proteogenomics to Inform Cancer Diagnosis and Treatment. Cell 184, 1661–1670. 10.1016/j.cell.2021.02.055 33798439 PMC8459793

[B22] Rosenbaum P. Tchitchek N. Joly C. Rodriguez Pozo A. Stimmer L. Langlois S. (2021). Vaccine Inoculation Route Modulates Early Immunity and Consequently Antigen-specific Immune Response. Front. Immunol. 12, 645210. 10.3389/fimmu.2021.645210 33959127 PMC8093451

[B23] Slingluff C. L. Jr. Petroni G. R. Chianese-Bullock K. A. Smolkin M. E. Hibbitts S. Murphy C. (2007). Immunologic and Clinical Outcomes of a Randomized Phase II Trial of Two Multipeptide Vaccines for Melanoma in the Adjuvant Setting. Clin. Cancer Res. 13, 6386–6395. 10.1158/1078-0432.ccr-07-0486 17975151

[B24] Stanfield B. A. Pahar B. Chouljenko V. N. Veazey R. Kousoulas K. G. (2017). Vaccination of Rhesus Macaques with the Live-Attenuated HSV-1 Vaccine VC2 Stimulates the Proliferation of Mucosal T Cells and Germinal center Responses Resulting in Sustained Production of Highly Neutralizing Antibodies. Vaccine 35, 536–543. 10.1016/j.vaccine.2016.12.018 28017425

[B25] Stanfield B. A. Rider P. J. F. Caskey J. Del Piero F. Kousoulas K. G. (2018). Intramuscular Vaccination of guinea Pigs with the Live-Attenuated Human Herpes Simplex Vaccine VC2 Stimulates a Transcriptional Profile of Vaginal Th17 and Regulatory Tr1 Responses. Vaccine 36, 2842–2849. 10.1016/j.vaccine.2018.03.075 29655629

[B26] Stanfield B. A. Stahl J. Chouljenko V. N. Subramanian R. Charles A.-S. Saied A. A. (2014). A Single Intramuscular Vaccination of Mice with the HSV-1 VC2 Virus with Mutations in the Glycoprotein K and the Membrane Protein UL20 Confers Full Protection against Lethal Intravaginal Challenge with Virulent HSV-1 and HSV-2 Strains. PLoS One 9, e109890. 10.1371/journal.pone.0109890 25350288 PMC4211657

[B27] Stransky N. Cerami E. Schalm S. Kim J. L. Lengauer C. (2014). The Landscape of Kinase Fusions in Cancer. Nat. Commun. 5, 4846. 10.1038/ncomms5846 25204415 PMC4175590

[B28] Treat B. R. Bidula S. M. Ramachandran S. St Leger A. J. Hendricks R. L. Kinchington P. R. (2017). Influence of an Immunodominant Herpes Simplex Virus Type 1 CD8+ T Cell Epitope on the Target Hierarchy and Function of Subdominant CD8+ T Cells. Plos Pathog. 13, e1006732. 10.1371/journal.ppat.1006732 29206240 PMC5736228

[B29] Uche I. K. Fowlkes N. Vu L. Watanabe T. Carossino M. Nabi R. (2021a). Novel Oncolytic Herpes Simplex Virus 1 VC2 Promotes Long-Lasting, Systemic Anti-melanoma Tumor Immune Responses and Increased Survival in an Immunocompetent B16F10-Derived Mouse Melanoma Model. J. Virol. 95, e01359. 10.1128/JVI.01359-20 33177208 PMC7925097

[B30] Uche I. K. Kousoulas K. G. Rider P. J. F. (2021b). The Effect of Herpes Simplex Virus-Type-1 (HSV-1) Oncolytic Immunotherapy on the Tumor Microenvironment. Viruses 13, 1200. 10.3390/v13071200 34206677 PMC8310320

[B31] Vance R. E. Eichberg M. J. Portnoy D. A. Raulet D. H. (2017). Listening to Each Other: Infectious Disease and Cancer Immunology. Sci. Immunol. 2, eaai9339. 10.1126/sciimmunol.aai9339 28783669 PMC5927821

[B32] Zhang L. Wang W. Wang S. (2015). Effect of Vaccine Administration Modality on Immunogenicity and Efficacy. Expert Rev. Vaccin. 14, 1509–1523. 10.1586/14760584.2015.1081067 PMC4915566 26313239

