# Supplementary material for: Utility of a Recombinant HSV-1 Vaccine Vector for Personalized Cancer Vaccines
Source: Front Mol Biosci. 2022 Jan 26;9:832393. doi: 10.3389/fmolb.2022.832393 (PMC8826227; doi:10.3389/fmolb.2022.832393)
Supplement: Supplementary file 1 [file DataSheet1.docx]

| P1 | CAATTTCACCGCCCCAGCACCGTTACCACCGATAGCGTCCGGGCGCTTGGAGGATGACGACGATAAGTAGGG |
| --- | --- |
| P2 | GTGGCCAAGACGAGCCCGCGCATGCCAAGCGCCCGGACGCTATCGGTGGTAACGGTGCTGGGGCGGTGAAATTGCAACCAATTAACCAATTCTGATTAG |
| P3 | ACAGCCCTCCCGACCGACACCCCCATATCGCTTCCCGACCTCC |
| OVA gBlock | ACAGCCCTCCCGACCGACACCCCCATATCGCTTCCCGACCTCCGGTCCCGATGGCCGTCCCGTTGGTGCTGTTGCCTGATGAAGTCTCAGGCCTTGAGCAGCTTGAGAGTATAATCAACTTTGAAAAACTGACTGAATGGACCAGTTCTAATGTTATGGAAGAGAGGAAGATCAAAGTGTACTTACCTCGCATGAAGATGGAGGAAAAATACAACCTCACATCTGTCTTAATGGCTATGGGCATTACTGACGTGTTTAGCTCTTCAGCCAATCTGTCTGGCATCTCCTCAGCAGAGAGCCTGAAGATATCTCAAGCTGTCCATGCAGCACATGCAGAAATCAATGAAGCAGGCAGAGAGGTGGTMKRRTCAGMAKWGGYTRRWTGGTTGCAATTTCACCGCCCCAGCACCGTTACCACCGATAGCGTCCGGGCGCTTGGAGGATGACGACGATAAGTAGGG |

Table S1. Primers and sequences used in this study. OVA sequences to be fused with VP26 were synthesized by Integrated DNA Technologies (IDT, Coralville IA) as gBlocks.
